# Does Previous Septoplasty Affect Rhinoplasty Outcomes? A Comparative Analysis of Functional, Psychological and Self-Assessment Measures

**DOI:** 10.3390/jcm15207819

**Published:** 2026-10-10

**Authors:** Marcin Jadczak, Alexandru Valeriu Breazu, Cristian Valentin Toma, Dariusz Jurkiewicz, Sandra Krzywdzińska

**Affiliations:** 1 Department of Otolaryngology with Division of Cranio-Maxillo-Facial Surgery, Military Institute of Medicine—National Research Institute, 04-141 Warsaw, Poland; djurkiewicz@wim.mil.pl; 2 Department of Anatomy, Carol Davila University of Medicine and Pharmacy, 050474 Bucharest, Romania; alexandru-valeriu.breazu@umfcd.ro (A.V.B.); cristian.toma@umfcd.ro (C.V.T.)

**Keywords:** rhinoseptoplasty, septoplasty, previous septoplasty, ROE, SCHNOS, NOSE, quality of life

## Abstract

**Background:** Patient satisfaction after rhinoseptoplasty depends on both functional outcomes and subjective factors, including self-perception, self-esteem and psychological well-being. **Methods:** A prospective study was conducted in 75 patients qualified for rhinoseptoplasty as follows: 47 women, 62.7%, and 28 men, 37.3%, aged 18–63 years, M = 34.24, and SD = 10.53. Twenty-seven patients (36%) had undergone previous septoplasty, including seven (9.3%) who required auricular cartilage grafting, while 48 patients (64%) had no prior septal surgery. Validated the Rhinoplasty Outcome Evaluation (ROE), the Standardised Cosmesis and Health Nasal Outcomes Survey (SCHNOS) and the Nasal Obstruction Symp-tom Evaluation (NOSE) questionnaires were completed preoperatively and at 1 and 6 months postoperatively. Quality of life was assessed with the World Health Organisation Quality of Life-BREF (WHOQOL-BREF) and self-esteem with Self-Esteem Scale, both before and after surgery. Data were analysed using IBM SPSS 29.0, with α = 0.05. **Results:** The groups showed improvement in aesthetic outcomes. Previous septoplasty was associated with lower overall rhinoplasty satisfaction at one month and lower cosmetic satisfaction at one and six months compared with primary cases (i.e., higher SCHNOS- Cosmesis (SCHNOS-C) scores, on which higher values denote greater dissatisfaction). NOSE scores improved markedly in both groups, while quality of life and self-esteem increased significantly after surgery, regardless of surgical history. **Conclusions:** Rhinoseptoplasty was followed by significant improvement in nasal function, quality of life and self-esteem; in the absence of a non-operated control group, these changes represent observed pre- to postoperative change rather than proof of surgical causation. Previous septoplasty may be associated with less favourable early functional and aesthetic recovery, but it does not prevent meaningful postoperative improvement. These patients may require more detailed preoperative planning and counselling.

## 1. Introduction

Rhinoseptoplasty is one of the most frequently performed procedures in facial plastic surgery, aiming to improve both nasal function and aesthetic appearance. Patient satisfaction following rhinoplasty depends not only on objective surgical outcomes but also on subjective factors, including self-perception, self-esteem and psychological well-being. Consequently, the assessment of postoperative outcomes has increasingly incorporated patient-reported outcome measures alongside traditional functional evaluations.

Septoplasty is commonly performed to correct nasal septal deviation and improve nasal airflow. Prior septoplasty may alter the structural integrity of the nasal framework, affect the availability of septal cartilage for grafting and potentially influence both surgical planning and postoperative outcomes. Despite these considerations, the impact of previous septoplasty on patient-reported functional and psychological outcomes after rhinoplasty remains insufficiently investigated [1,2].

Understanding whether a history of septoplasty affects patients’ expectations, preoperative self-assessment, postoperative satisfaction, or functional improvement is important for surgical decision-making and patient counselling. Furthermore, previous surgical experiences may influence psychological perceptions of nasal appearance and treatment outcomes, potentially affecting overall quality of life and satisfaction after rhinoplasty.

The aim of this prospective study was to evaluate the influence of previous septoplasty on preoperative and postoperative self-esteem, psychological well-being and functional outcomes in patients undergoing rhinoseptoplasty. We hypothesised that a history of septoplasty may influence patient-reported functional, cosmetic and psychosocial outcomes following rhinoseptoplasty and should therefore be considered during preoperative assessment, surgical planning, patient counselling and postoperative outcome evaluation. The present study represents a secondary analysis of an existing dataset, focusing on research questions that were not addressed in the original publication [3]. In contrast to the previous analysis, which evaluated outcomes at 1 and 3 months postoperatively, the present study included an extended follow-up period with assessments performed at 1 and 6 months after surgery, allowing for evaluation of more sustained postoperative changes and longer-term patient-reported outcomes.

## 2. Materials and Methods

The present study is a secondary analysis of data derived from a previously established prospective cohort of patients undergoing rhinoseptoplasty reported previously in “Assessment of Functional and Aesthetic Results in Preservation and Structural Rhinoplasty” [3]. Building on the existing patient database, additional analyses were undertaken to address the objectives of the current study. The project was conducted in collaboration with the Carol Davila University of Medicine and Pharmacy, which provided scientific and methodological support throughout the study. Initially, 86 consecutive patients scheduled to undergo rhinoseptoplasty were enrolled. One participant withdrew prior to the baseline assessment, and three additional participants discontinued their participation during the follow-up period. Furthermore, seven patients were excluded from the final analysis because they failed to attend the scheduled postoperative follow-up visit within the predefined study period or did not complete the study questionnaires according to the study protocol. Consequently, complete datasets were available for 75 patients, who constituted the final study population. The final cohort comprised 47 women (62.7%) and 28 men (37.3%). Participants ranged in age from 18 to 63 years (mean ± SD, 34.24 ± 10.53 years). A positive history of previous septoplasty was identified in 27 patients (36%). In all patients with a history of septoplasty, rhinoseptoplasty was performed no earlier than 6 months after the initial procedure. Of these, 7 patients (9.3% of the entire cohort) required additional grafting material for reconstruction, for which auricular cartilage was used. The second group comprised 48 patients (64%) who had not undergone previous septoplasty. Each participant received detailed information about the course of the study in both oral and written form and subsequently gave informed consent to participate. The study protocol was approved by the competent Bioethics Committee of the Military Medical Chamber (Resolution No. KB 24/24). Approval was obtained on 7 May 2024. To delineate the present manuscript from the primary publication, Table 1 summarises the relationship between the two analyses. The source cohort is the same; the present analysis addresses a different research question (the influence of a history of septoplasty on patient-reported outcomes), applies a different follow-up structure (1 and 6 months versus 1 and 3 months in the original analysis) and reports endpoints that were not analysed previously, namely the between-group comparison of ROE and SCHNOS domains and the WHOQOL-BREF and self-esteem outcomes. All 75 patients analysed here belong to the cohort of the primary publication. The 3-month assessment collected for the primary publication was not analysed in the present study [3].

The study was conducted in patients who met the following inclusion criteria: impaired nasal patency and deformity of the external nose, age above 18 years, presence of a nasal hump, and no history of paranasal sinus disease, clinically diagnosed depression or body dysmorphic disorder (BDD). Patients with a history of previous septoplasty were eligible only if at least 6 months had elapsed since their previous surgical procedure to ensure complete postoperative healing and stabilisation of functional outcomes.

The exclusion criteria were: age below 18 years, a history of previous rhinoseptoplasty, the presence of nasal septal perforation, a history of cleft lip and palate, a history of orthognathic surgery, and excessively wide nasal bridge. Participants who did not consent to take part in the study, withdrew from follow-up or refused to complete the questionnaires were excluded from the study.

In all patients qualified for surgery, a comprehensive history was taken, and a meticulous preoperative assessment, medical examination and surgical planning were performed. Preoperative photographs and computer visualisation were obtained. The evaluation included the skin, the shape of the nose and its harmony with the face, as well as the internal nasal structures. The problem and the goals of the procedure were discussed individually and comprehensibly with each patient. Preoperative computed tomography (CT) of the nose and paranasal sinuses was not routinely performed, as CT imaging is not part of the standard preoperative work-up for rhinoplasty. CT was obtained selectively based on individual clinical indications, such as suspected chronic rhinosinusitis or other sinonasal pathology. When performed, CT findings were incorporated into the preoperative assessment and surgical planning. Patients in whom CT imaging revealed findings meeting any of the predefined exclusion criteria (e.g., chronic rhinosinusitis or septal perforation) were excluded from the study. Objective assessment of nasal airflow (rhinomanometry, acoustic rhinometry or peak nasal inspiratory flow) was not performed, because the participants did not consent to these additional examinations; functional outcomes were therefore assessed exclusively by means of validated patient-reported instruments.

The operations were performed under general endotracheal anaesthesia using an external (open) approach with an inverted-V columellar incision. All procedures were performed by the senior surgeon, who had more than 30 years of experience in rhinoseptoplasty surgery. After the procedure, septal stabilising splints, bilateral anterior nasal packing and an external thermoplastic splint dressing were applied in both groups. The anterior packing was removed 24 h after surgery. Patients received identical recommendations regarding cleansing of the nasal cavities with seawater preparations. The external dressing, skin sutures and septal stabilising splints were removed 7 days after surgery. Patients completed the questionnaires at the following three time points: before surgery (measurement 1), one month after surgery (measurement 2) and six months after surgery (measurement 3). The questionnaire set comprised the following standardised outcome measures: the Standardised Cosmesis and Health Nasal Outcomes Survey (SCHNOS) with SCHNOS-Obstruction (SCHNOS-O), SCHNOS- Cosmesis (SCHNOS-C) and SCHNOS TOTAL, the Rhinoplasty Outcome Evaluation (ROE) and the Nasal Obstruction Symptom Evaluation (NOSE). [4,5,6,7,8,9,10] In addition, participants assessed their quality of life using the World Health Organisation Quality of Lif-BREF (WHOQOL-BREF) questionnaire, completed before surgery and after surgery [9]. The questionnaire evaluated overall quality of life across domains, including physical quality of life, psychological quality of life, social quality of life and environmental quality of life. Self-esteem was assessed with a separate, validated instrument, the 10-item Rosenberg Self-Esteem Scale (RSES), in its Polish adaptation [11,12], and not with items derived from the WHOQOL-BREF. The RSES is rated on a four-point Likert scale with a total score ranging from 10 to 40, where higher scores indicate higher self-esteem. The direction of scoring differs between the instruments used as follows: on the ROE, higher scores denote greater satisfaction, whereas on both SCHNOS domains and on the NOSE, higher scores denote a greater symptom burden or greater dissatisfaction. A decrease in SCHNOS and NOSE scores therefore corresponds to clinical improvement, and an increase in ROE scores likewise corresponds to improvement [4,5,6,7,8,9,10].

### Statistical Analysis

Statistical analyses were performed using IBM SPSS Statistics 29.0. Basic descriptive statistics were calculated, together with the Shapiro–Wilk test to assess the conformity of the distribution with normal distribution. The Mann–Whitney U test was used to compare the two groups. The Wilcoxon test was used to compare two measurements, and the Friedman test was used when more than two measurements were compared. Dunn’s test with Bonferroni correction of the significance level was used as the post hoc test. For variables meeting the assumptions of normality of distribution and homogeneity of variance, a 2 × 2 mixed-design analysis of variance was performed, with the Bonferroni test used as the post hoc test. For the purposes of this report, the significance level was set at α = 0.05. Because the between-group analysis comprises twelve comparisons performed at α = 0.05, the family-wise error rate was considered. Under a Bonferroni correction the adjusted threshold would be α = 0.0042, which none of the observed *p* values reaches. The between-group comparisons are therefore reported as exploratory and are interpreted together with the corresponding effect sizes rather than on the basis of statistical significance alone. Effect sizes were expressed as r = Z/√N for the Mann–Whitney U and Wilcoxon tests and as partial eta squared for the analysis of variance (ηp^2^ = F × df1/(F × df1 + df2), where df1 and df2 denote the effect and error degrees of freedom); values of r ≈ 0.1, 0.3 and 0.5 and of ηp^2^ ≈ 0.01, 0.06 and 0.14 were interpreted as small, moderate and large effects, respectively. Because most outcome distributions were non-normal and were analysed with rank-based tests, results are reported as medians with interquartile ranges together with effect sizes rather than as confidence intervals.

## 3. Results

### 3.1. Effect of Previous Septoplasty on ROE, SCHNOS and NOSE Scores

Baseline demographic and clinical characteristics of the two groups are summarised in Table 2. Table 3 presents the comparative analysis of patients with and without a history of previous septoplasty with respect to ROE and SCHNOS scores (measurement 1—before surgery, measurement 2—one month after surgery, and measurement 3—six months after surgery). The analysis showed that patients with previous septoplasty obtained nominally significantly (unadjusted *p* < 0.05) lower ROE scores at the second measurement and higher SCHNOS-C scores at the second and third measurements (moderate effects). No between-group differences were found for the remaining variables. Importantly, the two groups did not differ at baseline on ROE or SCHNOS scores (all *p* ≥ 0.384, r ≤ 0.10), indicating a comparable preoperative functional and aesthetic burden. The previous-septoplasty group was, however, significantly older (39.5 ± 11.0 vs. 31.3 ± 9.3 years; *p* = 0.002), whereas the sex distribution did not differ (*p* = 0.301; Table 2). In view of the number of comparisons performed, these between-group findings should be regarded as exploratory (see Statistical Analysis Section).

Subsequently, the Friedman test was used to compare the scores across the three measurements separately in the groups of patients with a positive and a negative history of previous septoplasty. Statistically significant differences were demonstrated for all analysed variables in both groups. The post hoc analysis revealed statistically significant differences between all measurements for SCHNOS-O and SCHNOS Total in both groups, with the highest scores obtained at the first measurement and the lowest at the third. In the group without a history of previous septoplasty, statistically significantly lower ROE scores were found at the first measurement compared with the other two measurements and higher SCHNOS-C scores at the first measurement compared with the other two. In the group of patients with previous septoplasty, statistically significantly higher SCHNOS-C severity was found at the first measurement compared with the remaining measurements, whereas for ROE statistically significant differences were noted between all measurements; the highest scores were obtained at measurement 3 and the lowest at measurement 1. Detailed results are presented in Table 4.

The Wilcoxon test (Table 5) was used to compare NOSE scores across two measurements. Both the group without and the group with previous surgical experience obtained higher scores at measurement 1 (before surgery) than at measurement 3 (six months after surgery) (strong effects).

### 3.2. History of Previous Septoplasty, Quality of Life and Self-Esteem

Table 6 presents a summary of the results of the 2 × 2 mixed-design analysis of variance, in which the between-subject factor was history of previous septoplasty (group) and the within-subject factor was the two repeated measurements of quality of life or self-esteem. The detailed effects for the individual variables are discussed below.

Quality of life—physical domain. The analysis revealed a main effect of measurement: preoperative quality of life (M = 25.98; SE = 0.49) was rated significantly lower than postoperative quality of life (M = 29.34; SE = 0.41). The effects of surgical experience and of the interaction were not statistically significant. This means that quality of life was rated similarly in patients who had undergone previous surgery and in those who had not, regardless of the time of measurement.

Quality of life—psychological domain. The analysis revealed a main effect of measurement: quality of life in the psychological domain was rated significantly lower before surgery (M = 22.55; SE = 0.43) than after surgery (M = 25.59; SE = 0.32). The effects of surgery and of the interaction were not significant, indicating comparable ratings in both groups regardless of the time of measurement.

Quality of life—social domain. The analysis revealed a main effect of measurement, with lower ratings before surgery than after surgery. Neither the effect of surgical experience nor the interaction reached statistical significance.

Quality of life—environmental domain. The analysis revealed a main effect of measurement, with lower ratings before surgery than after surgery. The effects of surgical experience and of the interaction were not statistically significant.

Self-esteem. The analysis revealed a main effect of measurement, with self-esteem rated significantly lower before surgery than after surgery. The effects of surgical experience and of the interaction were not statistically significant, indicating that self-esteem improved comparably in both groups after surgery.

## 4. Discussion

Nasal plastic surgery is currently one of the most frequently performed facial procedures, undertaken by both plastic surgeons and otorhinolaryngologists [5,13,14,15]. The popularity of rhinoseptoplasty stems not only from the possibility of improving nasal aesthetics but also from its functional importance. Impaired nasal patency, post-traumatic deformities and congenital structural abnormalities can significantly affect patients’ quality of life, social functioning and psychological well-being [3,16].

Rhinoseptoplasty is regarded as one of the most demanding procedures in facial plastic surgery. This results from the need to simultaneously achieve a satisfactory aesthetic outcome and to preserve or improve nasal respiratory function. The success of the procedure depends not only on the surgeon’s anatomical knowledge, surgical experience and technical skills, but also on the ability to communicate appropriately with the patient. Identifying the patient’s expectations, assessing their motivation for treatment and realistically presenting the achievable results are of key importance. Misunderstanding the patient’s needs, or a discrepancy between expectations and the actual surgical result, may lead to dissatisfaction despite a technically correct procedure [8].

For this reason, a meticulous preoperative assessment together with precise surgical planning is an indispensable element of the treatment process. The initial consultation enables an objective evaluation of the patient’s health status, expectations, motivation and concerns regarding the planned procedure. It is particularly important to obtain information about comorbidities that may affect wound healing and recovery. A detailed history of previous nasal procedures and of aesthetic medicine treatments using fillers is also essential [17,18].

**Figure 1:**
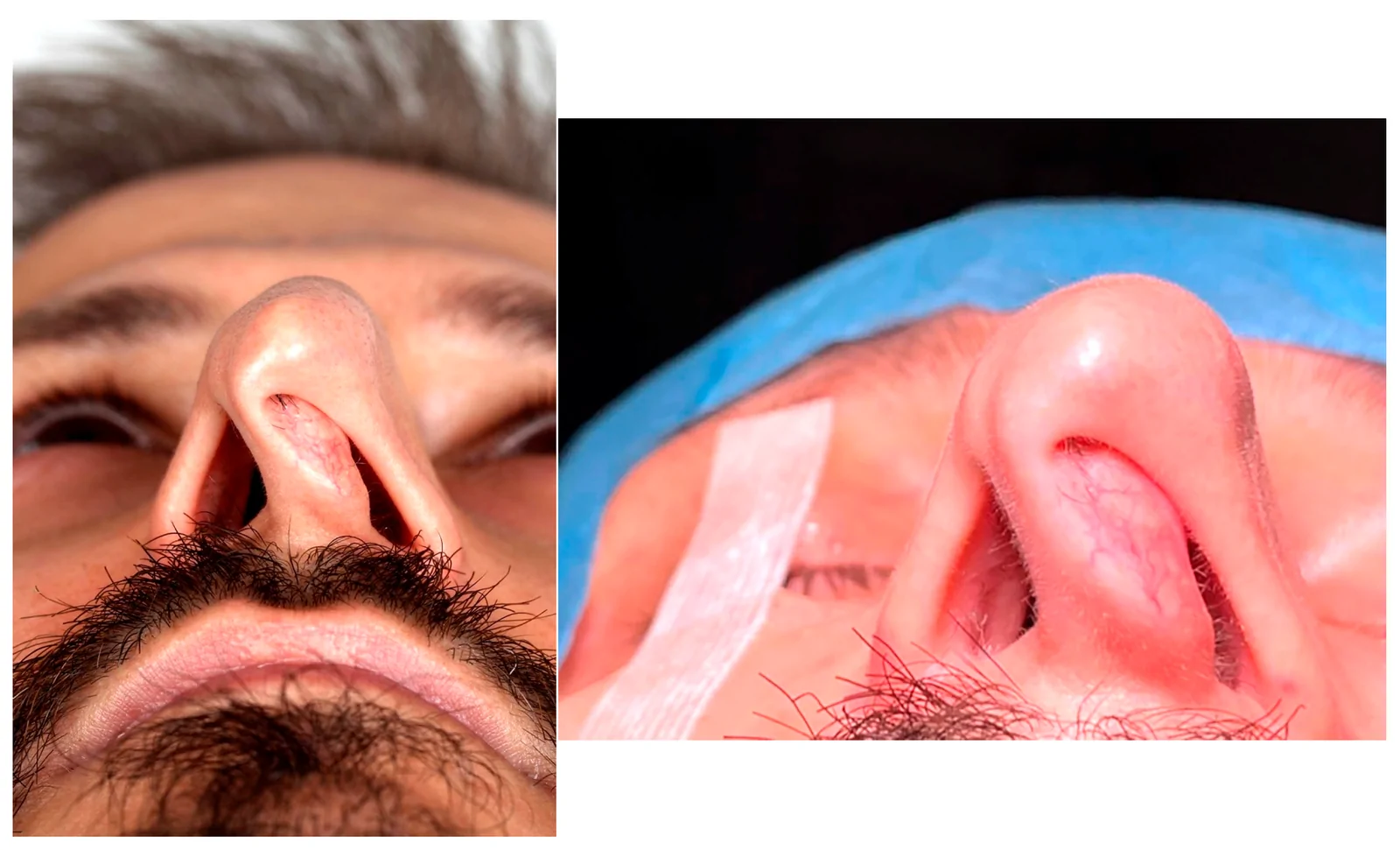
Preoperative view of a patient after previous septoplasty, demonstrating dislocation of the nasal columella.

**Figure 2:**
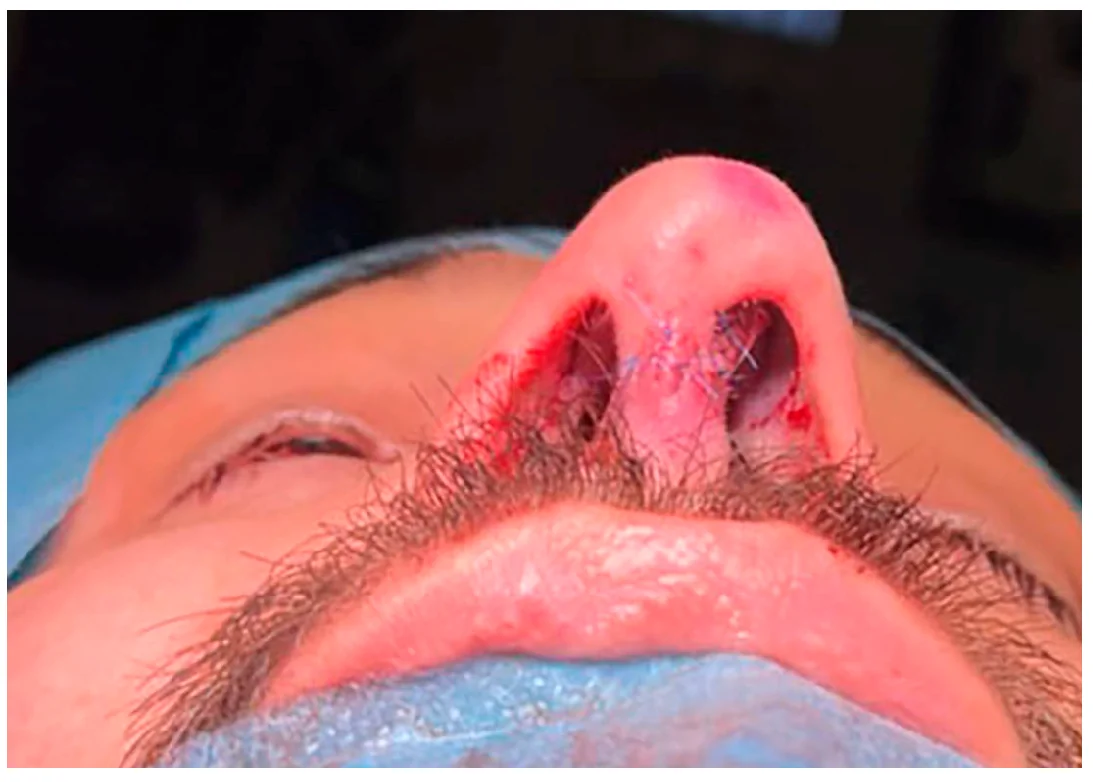
Immediate postoperative view of the patient after completion of rhinoseptoplasty.

**Figure 3:**
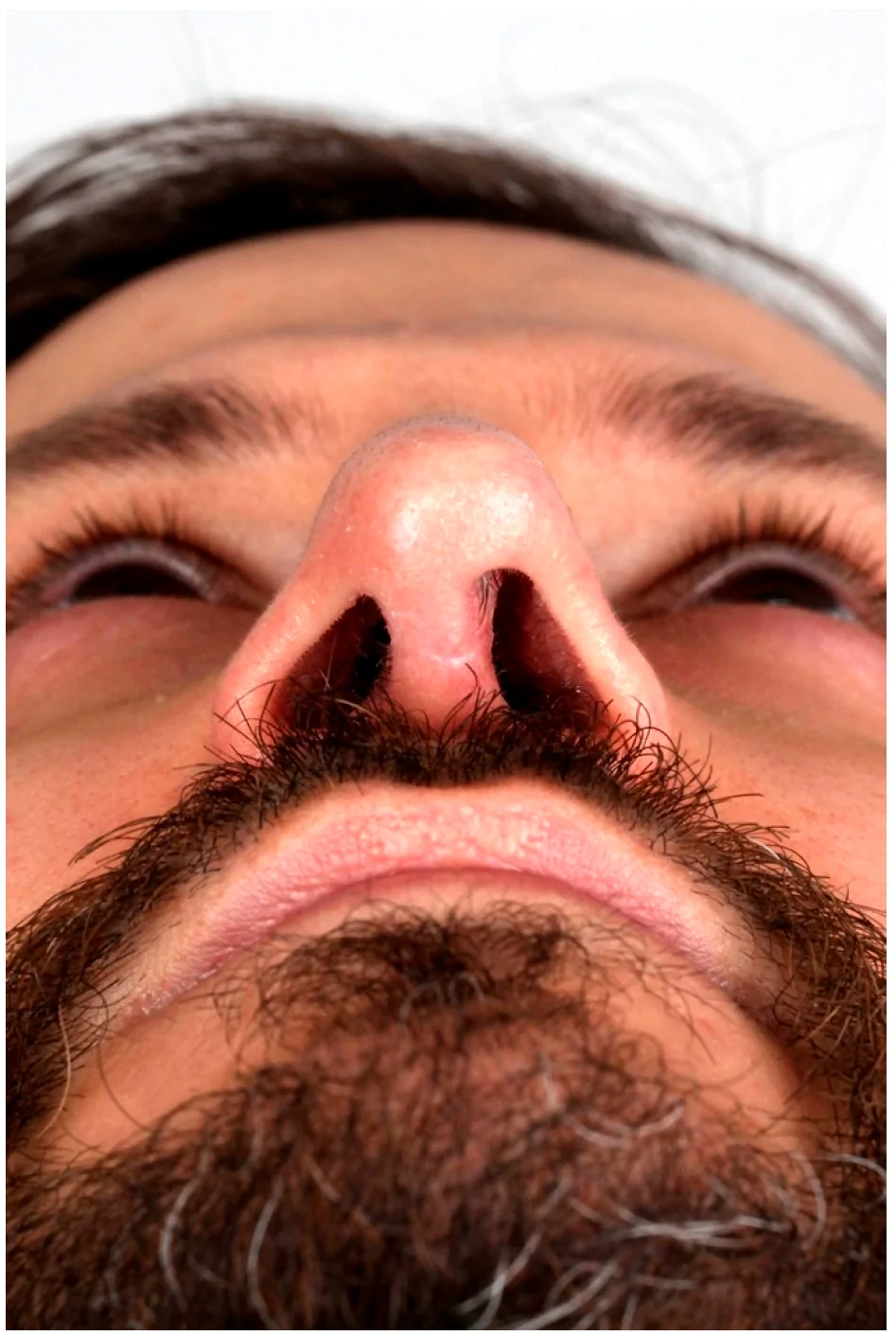
One-year postoperative view of the patient after rhinoseptoplasty.

Patients with a history of previous septoplasty constitute a particular group requiring an individualised diagnostic and therapeutic approach. This applies especially to those in whom, despite previous treatment, nasal patency disorders persist or symptoms recur after a period of initial improvement. Possible causes include incomplete correction of the primary septal deformity, secondary deviation of the septum during healing, nasal valve insufficiency, previously unrecognised deformities of the nasal skeleton, or excessive postoperative scarring of the tissues. These patients frequently report persistent difficulty in breathing through the nose, chronic obstruction, sleep disturbances and reduced quality of life despite previous surgical treatment. Consequently, they represent a group that is particularly demanding both diagnostically and therapeutically. A careful analysis of the causes of failure of the primary treatment and identification of all factors responsible for the persistence of symptoms are essential. Previous surgery also significantly increases the difficulty of a subsequent procedure [19]. The presence of scars, adhesions and altered anatomical relationships hinders tissue dissection and may limit the surgeon’s reconstructive options. Figure 1 shows a patient qualified for rhinoseptoplasty after a previously performed septoplasty; Figure 2 shows the same patient after completion of the operation and Figure 3 one year after the procedure.

Moreover, in many cases previous procedures lead to partial use or removal of the nasal septal cartilage, which is the primary grafting material used during rhinoseptoplasty. In such cases, it not infrequently turns out that the amount of cartilaginous material remaining in the nasal septum after the previous procedure is insufficient to perform rhinoseptoplasty. It then becomes necessary to use auricular or costal cartilage. This information should be discussed with the patient as early as the qualification stage, as it may affect both the extent of the operation and the course of recovery [20].

The psychological aspect is also of importance. Patients after previous septoplasty often approach a subsequent operation with a higher level of uncertainty and caution regarding the expected treatment results. At the same time, some of them associate very high expectations with the procedure, resulting from functional complaints that have persisted for months or even years. For this reason, a thorough discussion of the possible benefits, limitations and potential difficulties associated with a further nasal operation becomes particularly important. Appropriate patient qualification and a realistic presentation of the expected surgical outcomes are of key importance for achieving satisfactory results, both from the surgeon’s and the patient’s perspective [21].

A terminological clarification is warranted. The patients described here underwent a primary rhinoseptoplasty in the presence of a previous septoplasty and therefore occupy an intermediate position between a truly primary rhinoplasty and a revision rhinoplasty performed after a previous rhinoplasty. Wherever the terms “secondary” and “revision” appear in this manuscript, they denote a procedure carried out on a previously operated septum and not a repeat rhinoplasty; the present findings should not be extrapolated to revision rhinoplasty in the strict sense.

Of the patients studied, 27 had a history of previous septoplasty. One female patient with a history of previous septoplasty withdrew from the planned procedure on the day of surgery because of fear of further deterioration of nasal patency. These concerns arose from the fact that, after the previously performed septoplasty, she had experienced an increase in symptoms related to nasal obstruction. In seven cases in this group, it was necessary to use additional grafting material, for which the surgeon chose auricular cartilage. The analysis showed that patients with a history of previous septoplasty achieved poorer outcomes at the first postoperative follow-up (nominal *p* < 0.05; exploratory), as reflected by lower scores on the ROE questionnaire, which assesses aesthetic satisfaction, nasal function and psychosocial well-being. They also reported lower satisfaction with nasal appearance, as measured by the cosmetic domain of the Standardised Cosmesis and Health Nasal Outcomes Survey (SCHNOS-C). These results may stem from the fact that patients after previous septal surgery often present with more complex anatomical and functional problems than those undergoing primary treatment. Previous procedures may result in adhesions, scars, septal deformities, weakening of the supporting structures, or abnormal tissue healing. These changes increase the difficulty of the subsequent procedure and may limit the surgeon’s reconstructive options. The psychological aspect is also relevant. Patients who have previously undergone septoplasty often report persistent or recurrent problems with nasal patency despite prior surgical treatment. Consequently, they may associate higher expectations with rhinoseptoplasty regarding improvement in both breathing function and nasal appearance. Even when an objectively good surgical result is achieved, their subjective assessment of the treatment outcome may be more critical than in patients undergoing surgery for the first time. An interesting observation is that these differences were most evident at the first postoperative follow-up. It may be assumed that, at this stage, the assessment of results is still significantly influenced by postoperative oedema (which, owing to the previous surgery, may persist longer than after a primary procedure), the process of tissue remodelling and the not yet fully stabilised aesthetic and functional result. This, however, requires further studies covering a longer observation period. These data are compatible with, but do not prove, a faster recovery of overall satisfaction in patients without previous septal surgery.

It should be emphasised, however, that despite the poorer results obtained by patients with a history of previous septoplasty at the first postoperative follow-up, a significant improvement in both nasal function and satisfaction with nasal appearance compared with the preoperative period was recorded in both groups. Moreover, at subsequent observation points, the results indicated the achievement of satisfactory treatment outcomes regardless of previous septal surgery. It is also worth noting that the recovery process after rhinoseptoplasty is prolonged and dynamic. The resolution of soft-tissue oedema, remodelling of scars and adaptation of the nasal supporting structures may take many months. Therefore, the results obtained at early postoperative follow-ups should be interpreted with caution, especially in patients with previous nasal surgery [22].

The persistence of the between-group difference in SCHNOS-C at six months, in contrast to the attenuation of the ROE difference, deserves separate comment. Two non-exclusive explanations are plausible. The first is anatomical: previous septal surgery depletes the septal cartilage available for grafting, leaves scarring within the soft-tissue envelope and alters the mechanical behaviour of the skin, all of which may constrain the precision of dorsal and tip refinement that patients evaluate when rating the appearance of the nose. The second is psychological: patients who have already undergone an operation that did not fully resolve their complaints may be presensitised to nasal outcomes and may apply a more exacting internal standard when judging appearance, independently of the objective result. The present design cannot separate these mechanisms, because neither blinded aesthetic assessment nor psychometric measurement of preoperative expectations was available; doing so would require a study incorporating independent aesthetic rating and expectation measures alongside the patient-reported instruments.

Within the previous septoplasty group, the seven patients who required auricular cartilage grafting constitute the anatomically most demanding subgroup. Descriptively, all seven achieved improvement in both nasal function and satisfaction with the appearance of the nose relative to baseline and no donor-site complications were recorded. With *n* = 7, however, any formal comparison against the remaining twenty patients of this group would be substantially underpowered; we therefore refrain from inferential testing and report this subgroup descriptively only.

In the authors’ view these observations have important practical implications. Patients with a history of previous septoplasty should be appropriately informed that the process of achieving the full effect of the operation may take longer than in patients undergoing surgery for the first time. Proper preparation of the patient for the course of recovery and a realistic discussion of the expected results may contribute to increased treatment satisfaction and reduce unwarranted concerns in the early postoperative period. In concrete terms, patients with a previous septoplasty can be told that, in this cohort, the difference in overall satisfaction was present at one month and no longer detectable at six months, whereas the difference specific to the appearance of the nose was still present at six months. Scheduling an additional review between the first and sixth month and explicitly revisiting aesthetic expectations at the six-month visit are simple measures that follow directly from these data.

The quality-of-life analysis showed a significant pre- to postoperative improvement in all assessed domains of the WHOQOL-BREF questionnaire: physical, psychological, social and environmental. These domains encompass patients’ perceptions of their physical health, emotional well-being, body image and self-worth, interpersonal relationships and social support, as well as environmental factors such as personal safety living conditions, access to healthcare and opportunities for leisure activities. Comparison of preoperative and postoperative WHOQOL-BREF scores enabled assessment of the change in patients’ overall quality of life and its individual dimensions. Regardless of the patients’ previous surgical experience, quality of life after surgery was rated higher than before the procedure, indicating a broad multidimensional improvement in patients’ daily functioning after rhinoseptoplasty. The improvement in the physical domain including, among others, energy levels, comfort of functioning, sleep quality and the ability to perform daily activities, appears to be of the greatest clinical importance. The results are consistent with the possibility that improved perceived nasal patency after surgery translates not only into a reduction in local symptoms but also into patients’ overall physical well-being. A significant improvement was also recorded in the psychological domain. This result may indicate that correction of nasal deformities and improvement of respiratory function are associated with better self-perception of appearance, the sense of attractiveness, self-confidence and overall psychological well-being. It is worth emphasising that the nose occupies a central place in facial anatomy and plays an important role in shaping body image. For this reason, even relatively minor aesthetic changes can lead to a noticeable improvement in satisfaction with appearance and in psychosocial functioning. The improvement in quality of life in the social domain suggests that the positive effects of treatment extend beyond purely medical aspects. Better self-esteem, greater self-confidence and reduced discomfort related to nasal appearance may promote improved interpersonal relationships, greater social activity and greater comfort in interactions with others. This is particularly important for patients who, before surgery, reported dissatisfaction with the appearance of the nose or limitations resulting from chronic nasal obstruction. A significant increase in quality of life after surgery was also recorded in the environmental domain. Although this area is less directly related to nasal function and aesthetics, it includes, among others, a sense of security, the ability to carry out daily activities, living conditions and overall satisfaction with functioning in one’s environment. These results may indicate that the improvements reported by patients after surgery are comprehensive and concern many aspects of their lives.

Of particular interest, no significant differences were found between patients with a history of previous septoplasty and those undergoing surgery for the first time. This indicates that the pre- to postoperative change in quality of life was comparable regardless of previous surgical history. A similar relationship was observed for self-esteem where a significant increase in the level of self-esteem after surgery was found in both groups. These results are consistent with the possibility that improvement in the appearance and function of the nose contributes to increased self-worth regardless of previous surgical experience.

Taken together, the results indicate that patients showed measurable pre- to postoperative improvement not only in functional and aesthetic parameters, but also in terms of quality of life and psychological well-being in the broad sense. Importantly, the positive effects were observed regardless of the previous history of nasal surgery, which may constitute important information during patient qualification for the procedure and when discussing the expected treatment results with them. The results of the study conducted by the authors are consistent with the literature. For example, Kumar et al. [23], assessing patients over a one-year period (“The Use of Auricular Cartilage Grafts in Septorhinoplasty: A Dual-Centre Study of Donor Site Patient-Reported Outcome Measures”), reported a high level of satisfaction among patients undergoing rhinoseptoplasty with auricular cartilage used as grafting material. The authors demonstrated that harvesting cartilage from the auricle is associated with a low burden for the patient, both physically and psychologically, and that the functional and aesthetic effects obtained are rated very favourably. Importantly, most patients did not report significant complaints related to the donor site, nor a negative impact of the procedure on the appearance of the auricle or on daily functioning. These observations are of particular importance in the context of patients who have previously undergone nasal septal surgery. In this group, a deficiency of septal cartilage often limits reconstructive options and requires the use of alternative sources of grafting material. Our results, combined with the data available in the literature, suggest that the need to harvest additional auricular cartilage may not affect the final assessment of treatment outcomes by patients. Despite the greater extent of the surgical procedure, patients still achieve significant improvement in both nasal function and satisfaction with its appearance.

On the basis of the results obtained, it may be assumed that a previous septoplasty and the greater technical complexity of the procedure did not preclude satisfactory patient-reported results in this cohort. These results underline the importance of individualised surgical planning and a flexible approach to nasal reconstruction, tailored to the anatomical conditions and treatment history of each patient.

In the present study, no complications related to the auricular cartilage donor site were observed, such as auricular haematoma, wound infection, tissue necrosis or permanent ear deformity. This result is consistent with reports in the literature indicating that, with appropriate surgical technique, harvesting of auricular cartilage is a safe procedure associated with a relatively low risk of significant complications. One early postoperative complication was observed, in the form of early bleeding in a patient who had undergone previous septoplasty. This complication was recognised in the early postoperative period, and appropriate therapeutic management was implemented. The complication did not require reoperation. It did not significantly affect the final treatment result or the patient’s ultimate assessment of the functional and aesthetic effects. At present, revision surgery has not been required in any of the followed-up patients.

The low complication rate observed in this study may reflect both the safety of the cartilage-harvesting technique used and appropriate patient qualification for the procedure.

An equally important element of the treatment process is effective communication between the surgeon and the patient. A thorough discussion of the functional and aesthetic problems, presentation of the possible therapeutic options and a realistic definition of the expected surgical outcomes allow for a better understanding of the planned treatment and reduce the risk of discrepancy between the patient’s expectations and the achieved result. This becomes particularly important in the group of patients after previous nasal procedures, who because of persistent symptoms or unsatisfactory results of previous treatment often experience greater anxiety before a subsequent operation and have higher expectations regarding its results. At the same time, the diagnostic and therapeutic process in this group is usually more complex owing to the presence of postoperative changes and the reconstructive limitations resulting from previous surgical interventions.

The results obtained confirm that the effectiveness of rhinoseptoplasty should not be assessed solely on the basis of anatomical or functional parameters. Equally important are the patient’s subjective feelings, including satisfaction with nasal appearance, self-esteem and quality of life in the broad sense. In our study, improvement was recorded in all analysed areas, regardless of previous surgical history. Although patients with a history of previous septoplasty demonstrated poorer patient-reported outcomes during the early postoperative period, at the final follow-up the between-group difference in ROE scores was no longer detectable in this sample (*p* = 0.080). With 27 patients in the previous-septoplasty group, this result is as consistent with limited statistical power as with genuine convergence and does not establish equivalence between the groups; it indicates only that a difference could not be demonstrated at this time point. Patients with previous septoplasty, by contrast, continued to report lower satisfaction with the appearance of the nose, as reflected by persistently higher SCHNOS-C scores, on which higher values denote greater dissatisfaction.

In the present study, previous septoplasty did not preclude satisfactory functional, aesthetic and psychological outcomes after rhinoseptoplasty. It does, however, require more thorough preoperative planning, individualisation of the surgical approach and appropriate preparation of the patient for the course of recovery. Modern rhinoseptoplasty should therefore be regarded as a procedure requiring a holistic approach that takes into account not only the anatomical and technical aspects but also the patient’s psychological needs and individual expectations. It is precisely this combination of surgical precision with appropriate patient qualification and communication that constitutes the key to achieving lasting therapeutic success [2].

### Limitations

This study has several limitations that should be acknowledged. First, its observational design limits the ability to establish causal relationships between the investigated variables and the reported outcomes. Second, the assessment of functional outcomes relied exclusively on patient-reported outcome measures, as objective evaluation of nasal patency (e.g., rhinomanometry or acoustic rhinometry) could not be performed because the participants did not consent to these examinations. Consequently, the functional results reflect patients’ subjective perception rather than objective measurements of nasal airflow. In particular, the study included neither a non-operated control group nor a concurrent comparator, and the improvements reported in quality of life and self-esteem therefore describe an observed pre- to postoperative change within a treated cohort; they cannot be attributed causally to the operation itself. Regression to the mean, the natural course of symptoms, non-specific effects of undergoing treatment and the documented tendency of patient-reported instruments to improve after any intervention may all contribute to the magnitude of the observed change.

Another limitation is the absence of a blinded aesthetic evaluation by independent assessors. Postoperative aesthetic outcomes were evaluated solely by the patients using validated questionnaires, which may have introduced subjective bias. Furthermore, the follow-up period was limited to six months. Although this interval is generally considered sufficient to assess early functional and aesthetic outcomes after septorhinoplasty, longer follow-up is desirable to evaluate the stability of surgical results over time. Importantly, the study participants remain under routine clinical follow-up, allowing for the collection of additional long-term data in future analyses. This point deserves particular weight in rhinoplasty, where soft-tissue oedema resolves and scar tissue matures over a period that commonly extends to twelve months; the persistent SCHNOS-C difference observed here at six months may therefore attenuate or resolve by the one-year mark and the present data cannot exclude this possibility. Similarly, because no objective measurement of nasal airflow was available, every statement in this manuscript concerning functional outcome should be read as a statement about perceived nasal patency; whether the lower early satisfaction of previously operated patients reflects a genuine functional limitation or a difference in expectation cannot be determined from these data. The septoplasty group was significantly older than the non-septoplasty group (39.5 vs. 31.3 years; *p* = 0.002; Table 2) and age should therefore be considered a potential confounding factor when interpreting differences in aesthetic satisfaction. In addition, the severity of the initial septal deviation and of the external nasal deformity was not graded in a standardised way and the distribution of the surgical techniques (preservation vs. structural rhinoplasty) and of graft types between the groups could not be adjusted for in this sample; these factors may act as additional confounders. Finally, the between-group comparisons were numerous and unadjusted for multiplicity and the previous-septoplasty group comprised only 27 patients, so that null findings including the absence of group differences in quality of life and self-esteem should be interpreted as a failure to detect a difference rather than as evidence that no difference exists.

Despite these limitations, the study provides valuable real-world evidence regarding patient-reported functional, aesthetic and quality-of-life outcomes following septorhinoplasty using validated assessment instruments.

## 5. Conclusions

Rhinoseptoplasty was followed by a significant improvement in patient-reported nasal function, quality of life and patient self-esteem, regardless of a history of previous septoplasty. Patient-reported outcomes continued to improve between one and six months after surgery.

Patients who had previously undergone septoplasty may show lower overall and aesthetic satisfaction in the early postoperative period compared with those undergoing surgery for the first time. The between-group difference in overall ROE scores was no longer detectable at six months—a finding that reflects the absence of a demonstrable difference in this sample rather than proven equivalence—whereas the difference in SCHNOS-C scores, on which higher values denote greater dissatisfaction, persisted throughout the six-month follow-up. This suggests that previous septoplasty may affect primarily the course of recovery. The results obtained underline the importance of careful preoperative qualification, individualisation of the surgical approach and appropriate preparation of the patient for a potentially longer healing process in the case of previous nasal surgery. Nevertheless, further long-term studies and extended clinical follow-up are warranted to confirm these findings.

## Figures and Tables

**Table 1:** Relationship between the primary publication and the present secondary analysis.

Feature	Primary Publication	Present Secondary Analysis
Source cohort	Prospective cohort of patients undergoing rhinoseptoplasty	Identical source cohort; 75 patients with complete datasets
Grouping variable	Surgical technique (preservation vs. structural rhinoplasty)	History of previous septoplasty (present vs. absent)
Follow-up time points	Baseline, 1 month, 3 months	Baseline, 1 month, 6 months
Instruments reported	ROE, SCHNOS, NOSE	ROE, SCHNOS (O, C, Total), NOSE, WHOQOL-BREF, self-esteem
Endpoints unique to this analysis	—	Between-group comparison by septoplasty history; WHOQOL-BREF domains; self-esteem; 6-month patient-reported outcomes

**Table 2:** Baseline demographic and clinical characteristics of the two groups.

Variable	No Prior Septoplasty (*n* = 48)	Previous Septoplasty (*n* = 27)
Age, years (M ± SD)	31.29 ± 9.26	39.48 ± 10.98
Female, *n* (%)	28 (58.3%)	19 (70.4%)
Male, *n* (%)	20 (41.7%)	8 (29.6%)
Auricular cartilage graft required, *n* (%)	0 (0.0)	7 (25.9%)
ROE, baseline (Mdn, IQR)	1.08 (1.00)	1.17 (1.17)
SCHNOS-O, baseline (Mdn, IQR)	3.38 (1.25)	3.00 (2.50)
SCHNOS-C, baseline (Mdn, IQR)	3.83 (1.50)	3.83 (0.50)
SCHNOS Total, baseline (Mdn, IQR)	3.60 (0.90)	3.40 (0.90)
NOSE, baseline (Mdn, IQR)	3.20 (1.15)	3.40 (1.40)

M, mean; SD, standard deviation; Mdn, median; IQR, interquartile range.

**Table 3:** Comparison of ROE and SCHNOS scores between patients with and without a history of previous septoplasty.

	No Prior Septoplasty (*n* = 48)					Previous Septoplasty (*n* = 27)							
Dependent Variable	Mean Rank	M	SD	Mdn	IQR	Mean Rank	M	SD	Mdn	IQR	Z	*p*	r
ROE–measurement 1	36.36	1.11	0.59	1.08	1.00	40.91	1.29	0.76	1.17	1.17	−0.87	0.384	0.10
ROE–measurement 2	42.90	3.52	0.41	3.67	0.50	29.30	3.23	0.52	3.33	0.67	−2.62	0.009	0.30
ROE–measurement 3	41.25	3.64	0.35	3.75	0.50	32.22	3.49	0.42	3.67	0.50	−1.75	0.080	0.20
SCHNOS-O measurement 1	38.57	3.32	1.04	3.38	1.25	36.98	3.15	1.43	3.00	2.50	−0.30	0.761	0.04
SCHNOS-O measurement 2	37.46	1.08	0.58	1.00	0.75	38.96	1.19	0.77	1.25	1.25	−0.29	0.773	0.03
SCHNOS-O measurement 3	36.54	0.56	0.45	0.50	0.69	40.59	0.66	0.50	0.50	0.75	−0.79	0.432	0.09
SCHNOS-C measurement 1	37.47	3.70	1.19	3.83	1.50	38.94	3.86	0.84	3.83	0.50	−0.28	0.777	0.03
SCHNOS-C measurement 2	32.90	0.57	0.80	0.33	0.67	47.07	1.06	1.02	0.83	1.17	−2.73	0.006	0.32
SCHNOS-C measurement 3	32.99	0.45	0.66	0.17	0.50	46.91	0.81	0.81	0.67	0.83	−2.69	0.007	0.31
SCHNOS Total measurement 1	38.53	3.55	0.84	3.60	0.90	37.06	3.57	0.83	3.40	0.90	−0.28	0.778	0.03
SCHNOS Total measurement 2	34.85	0.77	0.54	0.60	0.60	43.59	1.11	0.80	0.90	1.10	−1.67	0.094	0.19
SCHNOS Total measurement 3	34.61	0.49	0.43	0.30	0.50	44.02	0.75	0.59	0.50	1.00	−1.81	0.070	0.21

M—mean; SD—standard deviation; Mdn—median; IQR—interquartile range; Z and *p* from the Mann–Whitney U test; r—effect size.

**Table 4:** Comparison of ROE and SCHNOS scores across measurements in patients with and without a history of previous septoplasty.

	Measurement 1		Measurement 2		Measurement 3				
Variable	Mdn	IQR	Mdn	IQR	Mdn	IQR	χ^2^	*p*	Post hoc
No prior septoplasty									
ROE	1.08	1.00	3.67	0.50	3.75	0.50	89.24	<0.001	1 < 2; 1 < 3
SCHNOS-O	3.38	1.25	1.00	0.75	0.50	0.69	88.72	<0.001	1 > 2; 1 > 3; 2 > 3
SCHNOS-C	3.83	1.50	0.33	0.67	0.17	0.50	85.64	<0.001	1 > 2; 1 > 3
SCHNOS Total	3.60	0.90	0.60	0.60	0.30	0.50	91.90	<0.001	1 > 2; 1 > 3; 2 > 3
Previous septoplasty									
ROE	1.17	1.17	3.33	0.67	3.67	0.50	50.96	<0.001	1 < 2; 1 < 3; 2 < 3
SCHNOS-O	3.00	2.50	1.25	1.25	0.50	0.75	48.26	<0.001	1 > 2; 1 > 3; 2 > 3
SCHNOS-C	3.83	0.50	0.83	1.17	0.67	0.83	50.13	<0.001	1 > 2; 1 > 3
SCHNOS Total	3.40	0.90	0.90	1.10	0.50	1.00	51.24	<0.001	1 > 2; 1 > 3; 2 > 3

Post hoc analysis by Dunn’s test with Bonferroni correction. The table indicates differences between measurements, with direction, for statistically significant differences (*p* < 0.05).

**Table 5:** Comparison of NOSE scores across two measurements in patients with and without previous surgical experience.

Dependent Variable	Measurement 1		Measurement 3				
	Mdn	IQR	Mdn	IQR	Z	*p*	r
NOSE—no prior septoplasty	3.20	1.15	1.50	0.65	−5.91	<0.001	0.62
NOSE—previous septoplasty	3.40	1.40	1.40	0.60	−4.55	<0.001	0.63

**Table 6:** Summary of the mixed-design analysis of variance for quality of life, self-esteem and surgical experience.

Model	Effects	F	df	*p*	ηp^2^
Model 1	Time (quality of life—physical)	174.94	1; 73	<0.001	0.71
	Group (previous septoplasty)	0.30	1; 73	0.584	0.01
	Interaction	1.26	1; 73	0.267	0.02
Model 2	Time (quality of life—psychological)	147.42	1; 73	<0.001	0.67
	Group (previous septoplasty)	0.95	1; 73	0.332	0.02
	Interaction	0.41	1; 73	0.525	0.01
Model 3	Time (quality of life—social)	35.04	1; 73	<0.001	0.32
	Group (previous septoplasty)	1.85	1; 73	0.178	0.03
	Interaction	0.09	1; 73	0.763	<0.01
Model 4	Time (quality of life—environmental)	41.69	1; 73	<0.001	0.36
	Group (previous septoplasty)	0.08	1; 73	0.783	<0.01
	Interaction	0.56	1; 73	0.456	0.01
Model 5	Time (self-esteem)	92.06	1; 73	<0.001	0.56
	Group (previous septoplasty)	0.96	1; 73	0.330	0.01
	Interaction	1.07	1; 73	0.304	0.01

F—F statistic; df—degrees of freedom; ηp^2^—partial eta squared (effect size), calculated as F × df1/(F × df1 + df2).

## Data Availability

The data presented in this study are available from the corresponding authors upon reasonable request. The data are not publicly available due to privacy.
